# Exon skipping in *IspE* Gene is associated with abnormal chloroplast development in rice *albino leaf 4* mutant

**DOI:** 10.3389/fpls.2022.986678

**Published:** 2022-11-08

**Authors:** Tingting Xu, Jingjing Zhang, Yiran Liu, Qiuxin Zhang, Weiyan Li, Yueling Zhang, Meifeng Wu, Tong Chen, Daochun Ding, Wenyi Wang, Zemin Zhang

**Affiliations:** State Key Laboratory for Conservation and Utilization of Subtropical Agro-Bioresources, Guangdong Provincial Key Laboratory of Plant Molecular Breeding, College of Agriculture, South China Agricultural University, Guangzhou, China

**Keywords:** rice, albino leaf 4 (al4), methylerythritol phosphate (MEP), exon skipping, *OsIspE*

## Abstract

The formation of leaf color largely depends on the components of pigment accumulation in plastids, which are involved in chloroplast development and division. Here, we isolated and characterized the rice *albino leaf 4* (*al4*) mutant, which exhibited an albino phenotype and eventually died at the three-leaf stage. The chloroplasts in *al4* mutant were severely damaged and unable to form intact thylakoid structure. Further analysis revealed that the candidate gene encodes 4-diphosphocytidyl-2-C-methyl-D-erythritol kinase (IspE), which participates in the methylerythritol phosphate (MEP) pathway of isoprenoid biosynthesis. We further demonstrated that the mutation at the exon-intron junction site cause alternative splicing factors fail to distinguish the origin of the GT-AG intron, leading to exon skipping and producing a truncated *OsIspE* in the *al4* mutant. Notably, disruption of *OsIspE* led to the reduced expression of chloroplast-associated genes, including chloroplast biosynthetic and translation related genes and photosynthetic associated nuclear genes (PhANGs). In summary, these findings reveal that *OsIspE* plays a crucial role in chloroplast biogenesis and provides novel insights into the function of CMK during chloroplast development in rice.

## Background

The chloroplast as semi-autonomous organelle, plays an important role in photosynthesis in higher plants (Liu et al., 2021). Defects in chloroplast development can cause abnormal chlorophyll accumulation, such as albino, light green, etiolation, green-revertible yellow, and striped leaves. Leaf color mutations generally affect photosynthetic efficiency in plants, leading to stunted growth even death. Leaf color is a common, easily identifiable phenotype that is considered ideal materials for studying the mechanisms of photosynthesis, chloroplast synthesis and degradation in plants.

Accumulating evidences has revealed that chlorophyll accumulation is regulated by many factors, such as temperature, RNA editing, and splicing. In rice, several pentatricopeptide repeat proteins (PPR) protein have been reported to modulate chloroplast development by regulating RNA editing and splicing, such as *OsPPR6* and *CDE4* (Tang et al., 2017; Liu et al., 2021). The *OsPPR6* gene participates in the editing of *ndhB* and splicing of *ycf3* transcripts, thereby regulating early chloroplast biogenesis in rice (Tang et al., 2017). The *cde4* mutant exhibits abnormal chloroplast development and albino seedlings under low temperature conditions, owing to a defect in transcript splicing of the chloroplast genes *rpl2*, *ndhA*, and *ndhB* (Liu et al., 2021). Other genes have also been shown to regulate chloroplast development in different plant species. For example, the *AtDPG1* gene encodes a putative chloroplast localized protein, and the *dep1* mutant shows a delayed pale-green phenotype in *Arabidopsis* (Liu et al., 2016a). *OsAL1* encodes a sole octotricopeptide repeat protein (OPR) protein, and regulates chloroplast development by coordinating the expression of chloroplast related genes in rice (Zhang et al., 2016). *OsAL2* encodes a chloroplast group IIA intron splicing facilitator, and the *al2* mutant shows an albino phenotype at the early developmental stage in rice (Liu et al., 2016b). *OsCSL1* encodes a MAPK kinase kinase22 (MKKK22), and interacts with OsMKK4. The mutation of *OsCSL1* resulted in a chlorosis seedling lethality phenotype (Liang et al., 2022).

Isoprenoids such as chlorophylls, carotenoids, cytokinins, and gibberellins, play an important role in membrane structure, growth, pathogen defense, and photosynthesis in plants (Rodrı´guez-Concepcio´n and Boronat, 2002; Bohlmann and Keeling, 2008). Its precursors are two five-carbon compounds, isopentenyl diphosphate (IPP) and dimethylallyl diphosphate (DMAPP). Two pathways are involved in the biosynthesis of IPP and DMAPP, including mevalonic acid (MVA) pathway in the cytoplasm and the 2-C-methyl-D-erythritol 4-phosphate (MEP) pathway in plastids. IPP and DMAPP generated by the MVA pathway are used for the synthesis of ubiquinone, sterols, and brassinosteroids (Pulido et al., 2012; Henry et al., 2018). The MEP pathway provides precursor molecules for phylloquinones, gibberellins, and other compounds. Seven enzymatic steps are involved in the MEP pathway (Liu et al., 2018). Genes that participate in the MEP pathway responsible for the leaf color phenotype have been identified in rice (Chen et al., 2018). For example, *IspF* encodes a 2-C-methyl-D-erythritol-2,4-cyclodiphosphate synthase, which is involved in the MEP pathway. A single nucleotide mutation in *IspF* caused a yellow-green leaf phenotype in rice (Huang et al., 2018). *IspE* encodes 4-diphosphocytidyl-2-C-methyl-D-erythritol kinase (CMK), and catalyzes 4-diphosphocytidyl-2-C-methyl-D-erythritol (CDP-ME) into 4-diphosphocytidyl-2-C-methyl-D-erythritol-2-phosphate (CDP-MEP). A missense mutation of *IspE* caused green-revertible yellow leaf phenotype in rice (Chen et al., 2018). Although few studies have revealed the roles of the MEP pathway during chloroplast growth and development, knowledge of the underlying molecular mechanisms remains largely limited.

In the present study, we isolated and characterized a rice mutant *albino leaf 4* (*al4*). The MutMap+ method revealed that the candidate gene encodes IspE, a key enzyme in the MEP pathway. Furthermore, a mutation at the exon-intron junction site in *OsIspE* led to exon skipping and produced truncated *OsIspE* in the mutant. Several genes related to chlorophyll synthesis and chloroplast development were significantly down-regulated in the *al4* mutant. Our findings suggest that *OsIspE* is essential for chloroplast biogenesis and development in rice.

## Materials and methods

### Plant materials and growing conditions

The rice (*Oryza sativa* L.) *albino leaf 4* (*al4*) mutant identified from EMS (Ethyl methyl sulfonate) mutant lines with *Xian/Indica* rice cultivar Ruanhua2B (RH2B) background. Wild-type (RH2B) and *al4* mutant plants were cultivated in the experimental field, located in South China Agricultural University (Guangzhou), during the natural growing season. To examine the albino phenotype of the mutant clearly, the rice plants were grown in the greenhouse under a 12-h-light/12-h-dark cycle at 30°C for laboratory work. No significant differences were observed between *al4* mutants grown in the greenhouse and those grown in the experimental field.

### Pigment measurement

Pigment contents of seedlings were measured based on the method of Zhang et al. (2016) with slight modifications. Fresh leaves (approximately 0.1g) were collected and placed in a 10 mL centrifuge tube with 2 mL extraction buffer (ethanol: propanol: H_2_O=4.5:4.5:1) for 48 h in the dark at 4°C. After centrifuging at 12000 rpm for 5 min, the samples were analyzed measuring the absorbance at 470, 645, and 663 nm. Pigment contents were calculated using the following equations:


$$\text{ChlorophyII a}(\text{mg}\cdot {\text{g}}^{-1})=(12.21{\text{A}}_{663}2.81{\text{A}}_{645})\times \text{W}$$

$$\text{ChlorophyII b}(mg\cdot g^{-1})=(20.13{\text{A}}_{645}5.03{\text{A}}_{663})\times \text{W}$$

$$\text{Carotenoid}(\text{mg}\cdot {\text{g}}^{-1})=(1000{\text{A}}_{470}2.05\times \text{Chla}114\times \text{Chlb})/245\times \text{W}$$

### Electron microscopy

The second leaves of the wild-type and *al4* mutant plants were cut into small pieces for scanning electron microscopy observation. The pieces were fixed with 2.5% glutaraldehyde in a phosphate buffer (0.1 M, pH 7.4) for 24 h at 4°C. Washed with 0.1 M phosphate buffer for four times, the samples were incubated in 1% (v/v) OsO4 for 4 h. After staining with 1% uranyl acetate overnight, the samples were dehydrated using an ethanol series (30, 50, 70, 85, 95, and 100% ethanol solution) at room temperature, and subjected to incubation of the leaves at each concentration for 15 min. Following the gradient series of epoxy resin (acetone: resin=3:1, 1:1, 1:3, v/v), the samples were infiltrated at each concentration for 3 h, and then the mixtures were incubated in pure Epon812 for 12 h. Subsequently, the samples were embedded in paraffin and cut into thin 60-80nm sections using a microtome. The sections were observed using a transmission electron microscope (Leica EM UC6).

### MutMap+ for cloning the *OsIspE* gene

MutMap+ method was used to map *OsIspE* gene according to Fekih et al. (2013). Briefly, the bulk DNA used for MutMap+ analysis was prepared by mixing DNA of 50 green seedlings (Pool WT) and 50 albino seedlings (Pool MT) from the M_4_ generation. Subsequently, both bulk DNA samples were subjected to whole-genome sequencing. High-quality clean sequence reads of the mutant pool and WT pool were aligned to the reference genome RHB to enable the identification of SNPs, and the SNP-index and ΔSNP-index were calculated based on the SNP information.

### RNA extraction and RT−qPCR

Total RNA was extracted with the RNA extraction kit (TRIgol reagent) (DingGuo, Beijing) based on the manufacturer’s protocol. First strand of cDNA was synthesized using One-step gDNA Removal and cDNA Synthesis SuperMix (TransGen Biotech, Beijing). Quantitative real-time PCR (RT-qPCR) was conducted using the CFX ConnectTM real-time PCR system (Bio-Rad) with SYBR Green Pro Taq HS (AG, GuangZhou) according to the manufacturer’s instructions. The procedure was as follows: 95°C for 2 min, followed by 40 cycles at 95°C for 15 s, 60°C for 30 s and 72°C 30 s, finally extension for 5 min at 72°C. The rice *OsUBQ* gene was used as an internal control. All primers used in RT−qPCR are listed in **Table S1**.

### Plasmid construction and transformation

To generate *OsIspE*-cas9 lines, two target sites in the first and sixth exons were designed by CRISPR-GE website online. First target of a 20-bp nucleotide sequence in the first exon was “TGGCTTGCTCCACCCACCTC”, and second target sequence in the sixth exon was “AAGAAGGTCCCTACTGGTGC” (**Figure S1**). Two sgRNA expression cassette driven by the Zea mays U6a and U3 promoters respectively. Using Golden Gate ligation, two sgRNAs with different target sequences of *OsIspE* were constructed into a pYLCRISPR/Cas9Pubi-H vector according to the previous methods (Ma et al., 2016), and then transferred into ZH11 by *Agrobacterium tumefaciens-*mediated transformation. To examine the subcellular localization of the OsIspE protein, the coding region without the stop codon of *OsIspE* was obtained by PCR from the cDNA of the wild-type (RH2B). The amplified sequences were inserted into the C-terminus of GFP under the control of the Ubi and 35S promoter to generate the pRTV-OsIspE-cGFP and p1305-OsIspE-cGFP vectors. The primers used for vector construction are listed in **Table S2**.

### Subcellular localization in rice protoplasts

Rice seeds (ZH11) were treated with 75% ethanol for 1 min, washed with sterile H_2_O and 5% NaClO for 30 min, and then washed with sterile H_2_O for four times. The sterilized seeds were placed on 1/2×MS medium and grown in a greenhouse under a 16-h-light/8-h-dark cycle at 30°C for 12~14 days. Rice protoplast isolation was based on the method described by Zhang et al. (2011) with minor modifications. The leaves the seedlings were cut into 0.5 mm strips and submerged in 0.6 M mannitol for 20 min. Afterward, the mannitol solution was replaced with a 10-20 mL enzyme solution and then incubated for 4 h in the dark with gentle shaking at ambient temperature. The enzyme solution was carefully removed and 20 mL W5 medium was added. This was gently shaken for 30 min to release the protoplasts. The protoplasts were filtered through 40 μm nylon meshes into a 50 mL centrifuge tube and centrifuged at 1000 rpm for 10 min at an ambient temperature to collect the protoplast pellet. Thereafter, the protoplasts were washed twice with W5 medium, transferred to several 2 mL centrifugal tubes, and centrifuged at 1000 rpm for 5 min. Finally, 2-5 mL W5 was added to collect the protoplasts and prepared for DNA transfection. The pRTV-OsIspE-cGFP was transferred into rice protoplasts according to the method described by Liang et al. (2022). The samples were examined using a confocal microscopy (Leica TCS SP8).

## Results

### Phenotypic analysis of *albino leaf 4* mutant

**Figure 1:**
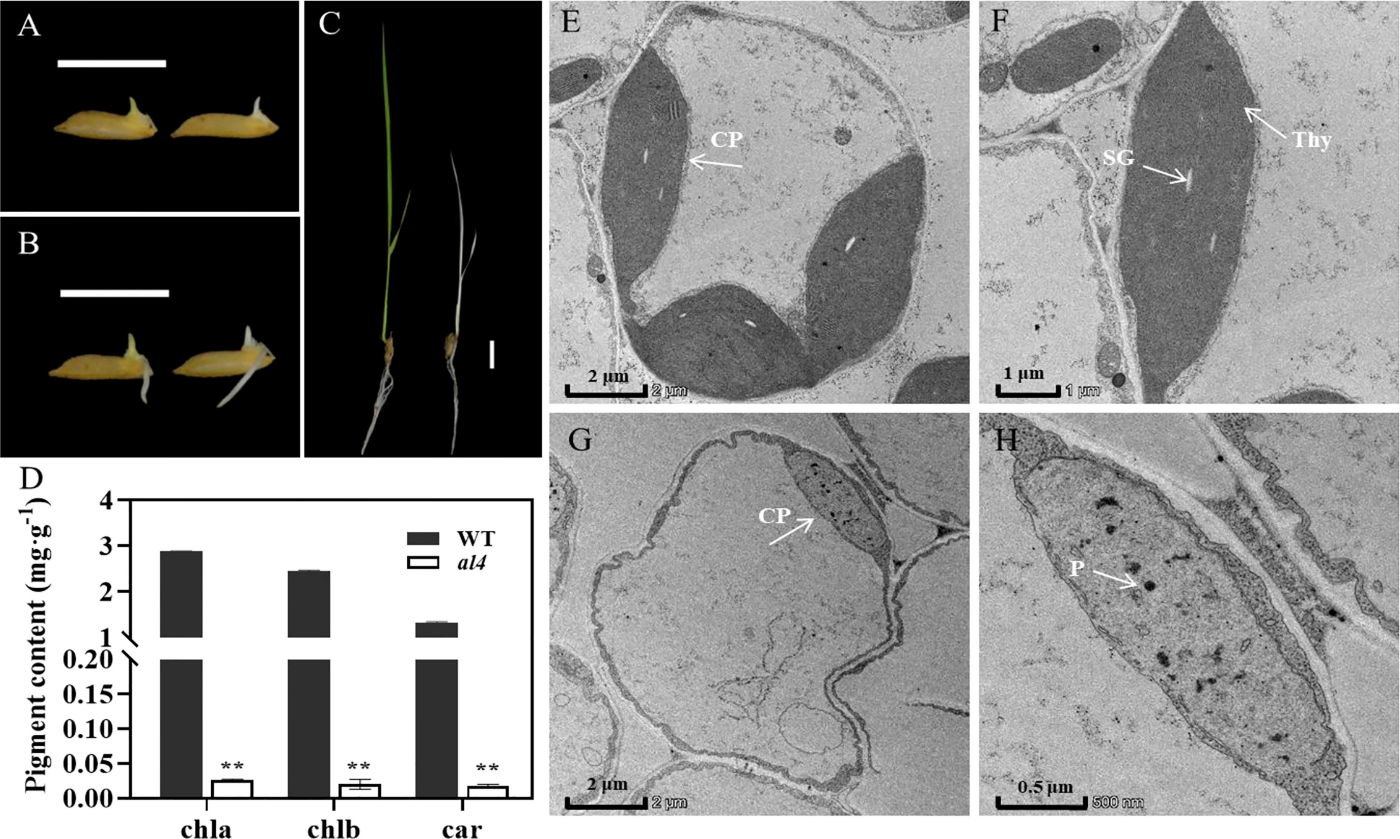
Phenotypic analysis of the *albino leaf 4* (*al4*) mutant. **(A–C)** Phenotypic comparison of WT (RH2B, left) and *al4* mutant (right) at 2 day **(A)**, 3 day **(B)** and 10 day **(C)** after germination (DAP). Bars=1 cm. **(D)** Chlorophyll (Chla, Chlb) and carotenoid (Car) levels between WT and *al4* mutant at 18 DAP. Data are presented as mean ± SD from three biological replicates. Two asterisks indicate statistically significant differences compared with the wild type at *P<*0.01. **(E–H)** Transmission electron microscopy analysis of the leaves in WT **(E, F)** and *al4* mutant **(G, H)** at the three-leaf stage. CP, chloroplast; SG, starch granule; Thy, thylakoid lamellar; P, plastoglobuli. Bar=2 μm **(E, G)**, 1 μm **(F)** and 0.5 μm **(H)**.

To investigate the underlying molecular mechanisms that participate in chloroplast development, an *albino leaf 4* (*al4*) mutant was isolated by genetic screening of EMS (Ethyl methyl sulfonate) mutant lines with *Xian*/*Indica* rice cultivar Ruanhua2B (RH2B) background. Phenotypic analysis showed that the buds and seedling in *al4* exhibited an albino phenotype compared to those in RH2B (wildtype, WT) (**Figures 1A–C**). Similar to other chlorotic or albino leaf mutants identified in our laboratory, such as *al1*, *al2*, and *csl1*, the seedlings gradually withered and eventually died (Zhang et al., 2016; Liu et al., 2016b; Liang et al., 2022). The albino phenotype in *al4* mutant also could not recover at later developmental stages and eventually died at the three-leaf stage.

Since the photosynthetic pigments chlorophylls and carotenoids are closely associated with capturing light energy in plants, we evaluated the chlorophyll (Chla, Chlb) and carotenoid (Car) content in *al4* mutant and WT plants. Consistent with the albino phenotype, *al4* had significantly lower Chla, Chlb and Car levels than WT (**Figure 1D**). These results suggest that the early-stage lethality phenotype in *al4* mutant might be caused by abnormal chloroplast development.

### *OsIspE* affects chloroplast development

Chlorophyll and carotenoid levels are generally modulated through chloroplast biogenesis in plants. Thus, transmission electron microscopy (TEM) was used to observe the chloroplast structure in *al4* and WT plants at the three-leaf stage. The results showed that the chloroplasts in WT plants exhibited abundant distinct thylakoids packed with grana and many starch granules (**Figures 1E, F**), whereas those of *al4* contained few undeveloped chloroplasts (**Figure 1G**). Plastoglobuli (PGs) were increased in *al4* mutant compared to WT (**Figure 1H**). The chloroplasts in *al4* were severely damaged and unable to form an intact thylakoid structure (**Figure 1H**). Collectively, these results suggest that the albino phenotype of *al4* is due to defects in chloroplast development.

### Molecular cloning of *OsIspE*

**Figure 2:**
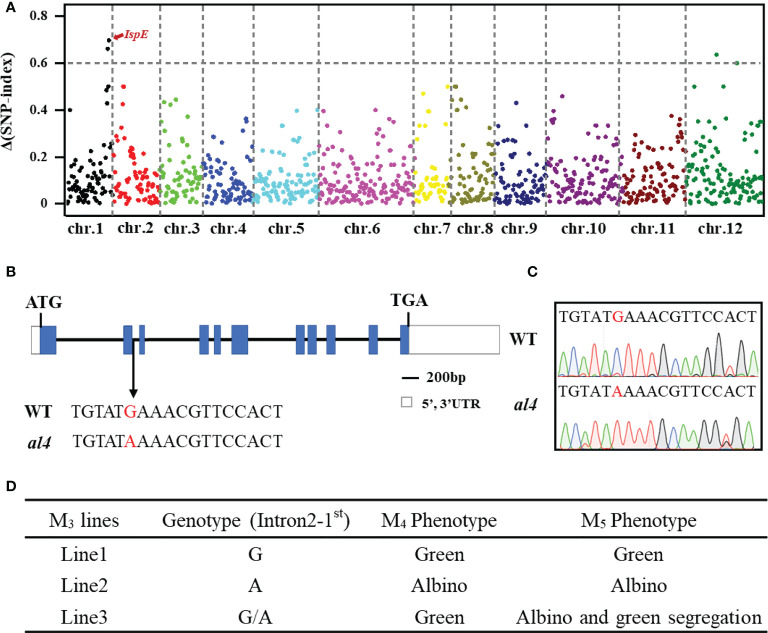
MutMap+ identifies the causal mutation of the *al4* early-stage lethality phenotype. **(A)** ΔSNP-index plot of the whole genome generated by MutMap+ analysis. **(B)** Schematic diagram of *al4* and point mutations between the WT and *al4* mutant. Blue boxes represent exons and black lines represent introns. **(C)** Genomic regions spanning the mutation site of *OsIspE* were amplified and sequenced. **(D)** Segregation verification in different EMS generations.

The segregation ratio of the green and albino seedlings in the heterozygous M_3_ plants was 284: 94 (χ^2^ = 0.0035, *P*>0.05, **Table S3**, **Figure S2**), indicating that the *al4* phenotype was controlled by a single recessive gene. The MutMap+ method was applied for rapid identification of the gene controlling the albino phenotype in the *al4* mutant, a bulk of 50 green seedlings (Pool WT) and 50 albino seedlings (Pool MT) from the M_4_ generation were separately aligned to the reference sequence. The SNP-index and ΔSNP-index were calculated with each SNP. The ΔSNP-index and chromosome positions are shown in **Figure 2A**. ΔSNP-index value over 0.35 were further analyzed, and a total of 41 SNPs were identified (**Table S4**). On the long arm of chromosome 1, a peak of the ΔSNP-index closes to 0.7 was observed (**Figure 2A**). In this candidate region, a G to A transition at the exon-intron junction site in *Os01g0802100* causes abnormal splicing of *OsIspE* (**Figures 2B, C**)

*Os01g0802100* encodes 4-diphosphocytidyl-2-C-methyl-D-erythritol kinase (CMK), which belongs to the IspE superfamily (Chen et al., 2018). We subsequently examined whether the albino leaf phenotype was consistent with the single deletion mutation in *OsIspE*, and the point mutation was verified in different EMS generations using genomic DNA sequencing. Leaf color phenotype segregation was observed in the M_5_ population seedling stage only the M_4_ plant genotype was heterozygous (**Figure 2D**; **Figure S3**), and the green/albino seedling segregation ratio in the M_5_ population was 94:28. Taken together, these results suggested that *Os01g0802100* is a strong candidate gene for *OsIspE*.

### Abnormal alternative splicing led to exon skipping in *al4*

**Figure 3:**
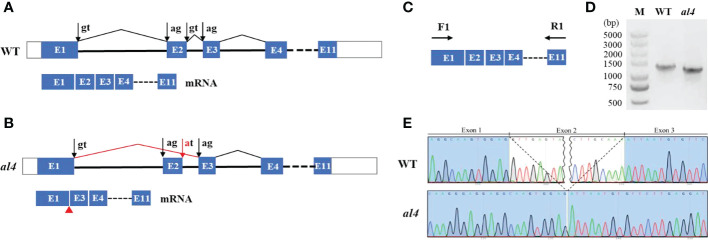
Intron mutation of *OsIspE* results in exon skipping. **(A)** Schematic representation of the structure of the WT genomic sequence of *OsIspE*. The positions of ‘‘canonical’’ splicing sites are shown with black arrows. **(B)** Exon-intron junction site mutation (red arrow), leading to exon skipping and production of truncated *OsIspE* in the *al4* mutant. **(C, D)** RT-PCR bands of *OsIspE* in the wild type and *al4* mutant with the specific primers indicated in panel C. **(E)** Chromatograms of cDNAs regions in the WT and *al4* mutant. The entire exon2 is absent in the *al4* mutant.

Alternative splicing is a common process in post-transcriptional mRNA regulation, that produces various mature mRNAs with different structures and functions. The full-length *OsIspE* gene consists of 1206 bp and including 11 exons (**Figure 3A**). To examine splicing alternation of *OsIspE* in the mutant, RT-PCR was performed with a forward and a reverse primer in exon1 and 11, respectively (**Figure 3C**). WT generated a 1206 bp fragment as predicted, whereas PCR product in *al4* mutant was smaller than that in WT (**Figure 3D**). To determine whether exon2 was missing in the *al4* mutant, a forward and a reverse primer in exon1 and 2 were designed. RT-PCR results showed that WT produced a 184 bp fragment as expected, but the *al4* mutant did not produce a fragment (**Figure S4**). Sanger sequencing revealed that 96 bp deletion and the entire exon2 was absent in the *al4* mutant (**Figures 3B, E**). This mutation resulted in the deletion of 32 amino acids in *al4* mutant (**Figures S5**, **S6**). RT-qPCR analysis of *OsIspE* expression in WT and *al4* revealed that *OsIspE* expression was significantly reduced in the *al4* (**Figure S7**). Protein structure prediction indicated that α-helix was include in the exon2 of OsIspE (**Figure S8**). Taken together, these results suggest that mutation at the exon-intron junction site cause alternative splicing factors fail to distinguish the origin of the GT-AG intron, leading to exon skipping and producing a truncated *OsIspE* in the *al4* mutant.

### *OsIspE* is responsible for the development of chloroplasts

**Figure 4:**
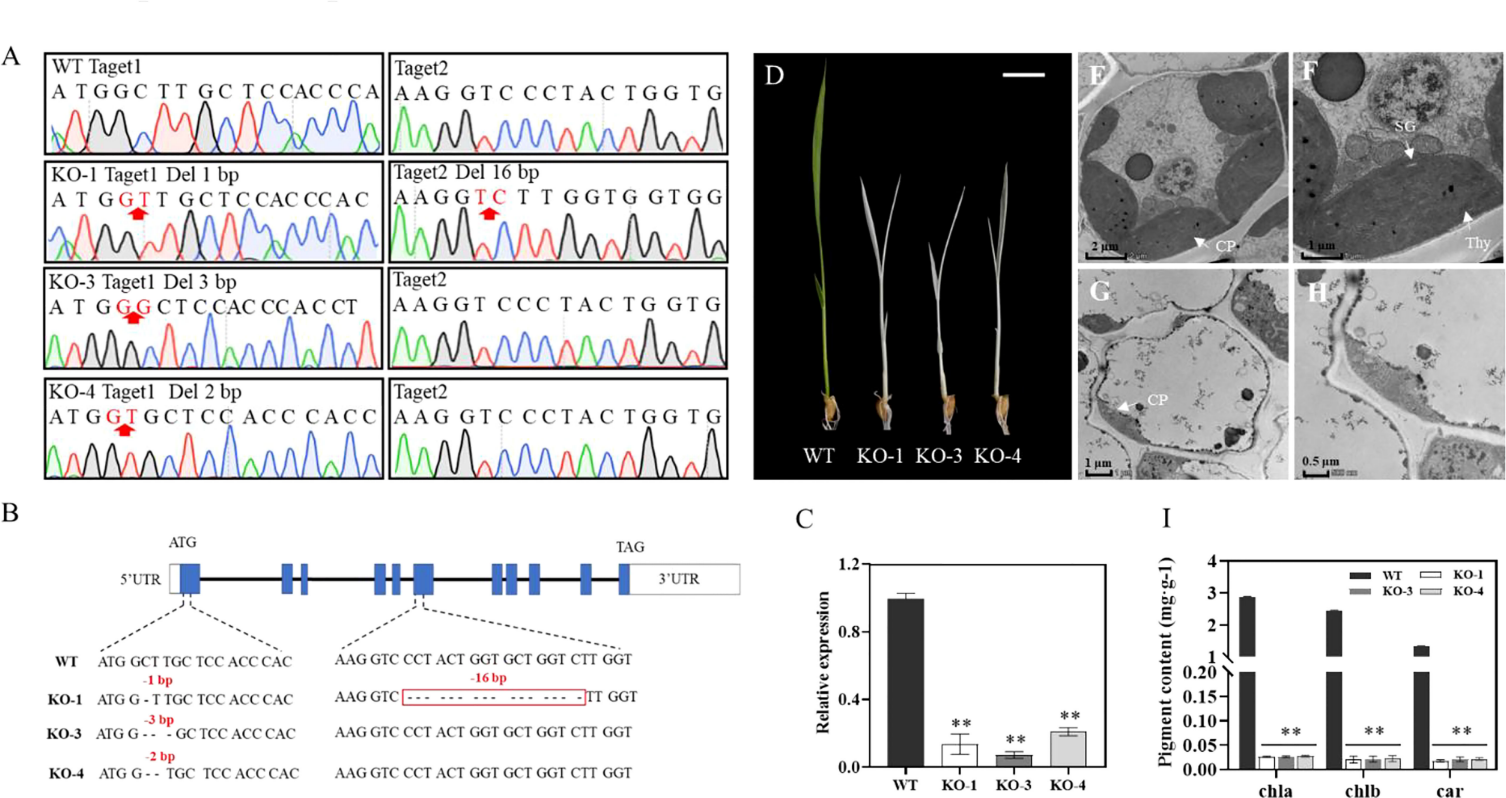
Transgenic confirmation of the role of *OsIspE* in regulating leaf color. **(A, B)** *OsIspE*-Cas9 lines (KO-1, KO-3, KO-4) containing deletion mutations were confirmed *via* sequencing. **(C)** RT-qPCR analysis of *OsIspE* in ZH11 and *OsIspE*-Cas9 lines. **(D)** Phenotypic analysis of *OsIspE-*Cas9 lines at 10 DAP. Bars=1cm. **(F–H)** Transmission electron microscopy analysis of the leaves of ZH11 **(E, F)** and KO*-*1 **(G, H)** at the three-leaf stage. CP, chloroplast; SG, starch granule; Thy, thylakoid lamellar. Bar=2 μm **(E)**, 1 μm **(F, G)** and 0.5 μm **(H, I)** Chlorophyll (Chla, Chlb) and carotenoid (Car) levels of ZH11 and *OsIspE*-Cas9 lines at 10 DAP. **P < 0.01.

To verify whether the mutation of *OsIspE* corresponded to the albino phenotype, we generated *OsIspE*-Cas9 transgenic plants by CRISPR/Cas9 genome-editing approach. Two target sites located on the first and sixth exon were selected and a total of 20 independent transgenic lines were obtained. Deletion mutations at target sites were characterized by DNA sequencing, and three *OsIspE*-Cas9 transgenic plants resulting in frameshift mutations were selected for further analysis (**Figures 4A, B**). In *OsIspE*-Cas9-1(KO-1) plants, 1bp and 16bp deletions were detected in target site 1 and 2, respectively (**Figure 4A**). In addition, 3bp and 2bp deletions in target site 1 were identified in KO-3 and KO-4 transgenic plants (**Figure 4A**). *OsIspE* expression levels were significantly decreased in the *OsIspE*-Cas9 lines compared to WT (**Figure 4C**). Phenotypic observation showed that *OsIspE*-Cas9 lines (KO-1, KO-3 and KO-4) displayed an albino leaf phenotype compared to WT (**Figure 4D**).

We further applied TEM to investigate the chloroplast structures of KO-1 and WT seedlings at the three-leaf stage. As expected, WT had well-developed chloroplasts, containing normally structured thylakoid lamellar and starch granule (**Figures 4E, F**), whereas chloroplasts in *OsIspE*-Cas9 plants displayed abnormal structures, without intact thylakoid lamellae (**Figures 4G, H**). We next measured the contents of Chla, Chlb and Car in WT and *OsIspE*-Cas9 lines, and the results showed that chlorophyll and carotenoid levels were significantly decreased in the *OsIspE*-Cas9 lines (**Figure 4I**). Taken together, these results demonstrated that *OsIspE* is essential for chloroplast development in rice.

### Expression pattern and subcellular localization of OsIspE

**Figure 5:**
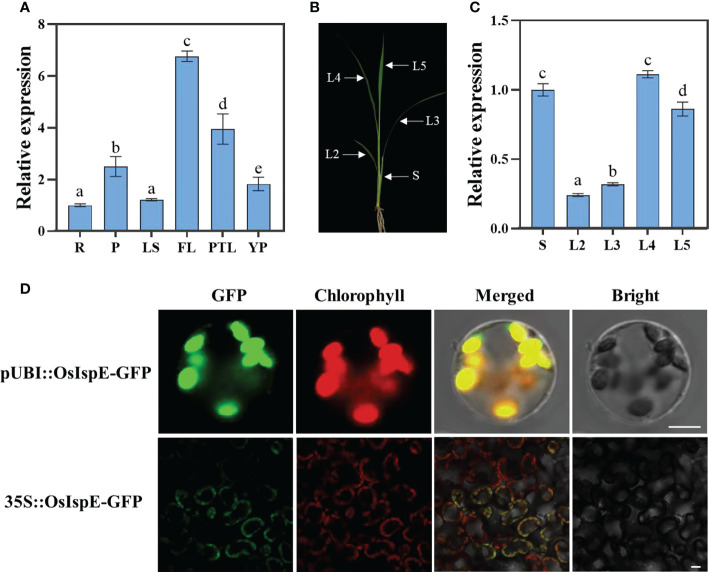
Expression pattern and subcellular localization of OsIspE. **(A)** *OsIspE* expression levels in different tissues. R, roots; P, pulvinus; LS, leaf sheaths; FL, flag leaves; PTL, pour two leaves; YP, young panicles. Values are means ± SD from three biological replicates. **(B)** Schematic of seedling plant used for RT-qPCR. S represent stem; L2-L5 represent the second to the fifth leaves. **(C)** *OsIspE* expression levels in different tissues of young seedlings. Values are means ± SD from three biological replicates. Data were analyzed using two-way ANOVA with *post hoc* Tukey tests (letters indicate significant differences between samples at *P*< 0.05). **(D)** Subcellular localization of OsIspE. pUBI::OsIspE-GFP and 35S::OsIspE-GFP were transiently transformed into rice protoplasts and tobacco leaves respectively. Fluorescence signals were detected using confocal microscopy, and representative images are shown. Bars=10 μm (rice protoplast) and 20 μm (tobacco leaves).

RT-qPCR was applied to detected transcript profiles of *OsIspE* in different tissues, including roots (R), pulvinus (P), leaf sheaths (LS), flag leaves (FL), pour two leaves (PTL), young panicles (YP). The results showed that *OsIspE* was expressed in different tissues and organs, and particularly abundant in flag leaf (**Figure 5A**). To further examined whether the expression of *OsIspE* corresponds to the period of early leaf development, we prepared the cDNA samples come from wild-type plants of 3‐week‐old seedlings (**Figure 5B**). And the results showed that *OsIspE* was expressed in all leaves, and preferentially expressed in the latest growing leaves, such as the L4 (**Figure 5C**). Notably, the *OsIspE* was highly expressed in stem.

To examine the subcellular localization of OsIspE, a transient expression vector harboring the OsIspE-GFP fusion protein was transiently transformed in rice protoplasts. The green fluorescent signals of OsIspE-GFP were co-localized with chlorophyll autofluorescence signals (**Figure 5D**), which consistent with the previous study (Chen et al., 2018). We further confirm the subcellular localization in tobacco leaves, and the fluorescence signals of OsIspE-GFP was also colocalized with chloroplast, suggesting that OsIspE is a chloroplast-localized protein.

### The mutation of *OsIspE* affected the expression of chloroplast-associated genes

**Figure 6:**
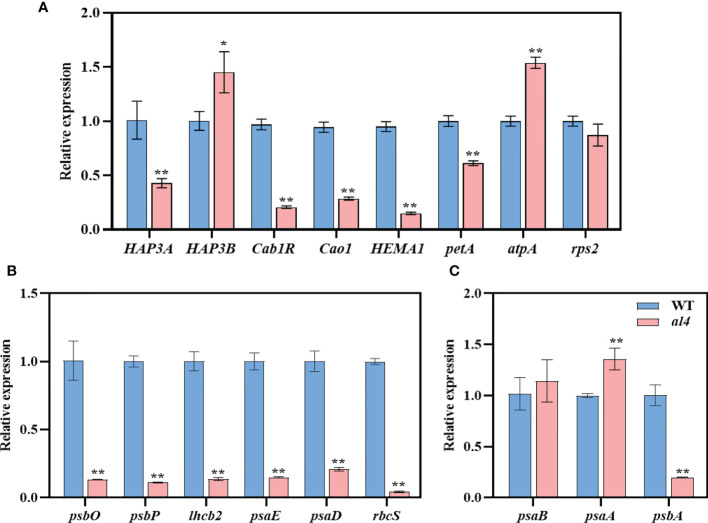
Expression of chloroplast-associated genes in WT and *al4.* Relative expression of chloroplast biosynthetic and translation related genes **(A)**, Photosynthetic associated nuclear genes (PhANGs) **(B)**, Plastid-encoded polymerases (PEPs)-dependent chloroplast genes **(C)**. Data are presented as mean ± S.D. *P < 0.05; **P < 0.01.

Since the *OsIspE* gene is involved in the MEP pathway, the mutation of *OsIspE* would cause an abnormal chloroplast structure. We assumed that the loss of *OsIspE* would influence the expression of chloroplast-associated genes. To examine this hypothesis, we investigated the expression of chloroplast-associated genes using RT-qPCR, including chloroplast biosynthetic and translation related genes, Photosynthetic associated nuclear genes (PhANGs) and plastid-encoded polymerases (PEPs)-dependent chloroplast genes. Compared to WT, the expression levels of several chloroplast biosynthetic and translation related genes were significantly reduced, such as *HAP3A*, *Cab1R*, *Cao1*, and *HEMAI* (**Figure 6A**). Chloroplast biosynthetic and translation related genes and PEP-dependent chloroplast genes displayed diverse expression patterns between WT and *al4* mutant, expression levels of *rps2* and *psaB* were not affected, *petA* and *psbA* were significantly reduced, and the *atpA* and *psaA* were upregulated in the *al4* compared to WT (**Figures 6A, C**). The expression levels of the PhANG genes, including *psbO*, *psbP*, *lhcb2*, *psaE*, *psaD*, and *rbcS* were dramatically reduced in the *al4* (**Figure 6B**). We also investigated the expression pattern of mitochondrial genes between WT and *al4* mutant, results showed several genes, such as *nad2*, *nad4*, and *nad5* were upregulated in the *al4* mutant (Fig S9). These results indicate that the mutation of *OsIspE* affects the expression of chloroplast-associated genes that regulate chloroplast development in rice.

## Discussion

Leaf color mutants are ideal materials for studying chlorophyll biosynthesis and chloroplast development in plants. Here, we isolated and characterized a *leaf albino 4* mutant using Ethyl methyl sulfonate (EMS) mutagenesis with *Xian/Indica* rice cultivar Ruanhua2B (RH2B) background. Further analysis revealed that the early-stage lethality phenotype in the *al4* mutant was caused by defective chloroplast development (**Figures 1G, H**). We used MutMap+ to identified that the mutation in *Os01g0802100* gene encoding 4-diphosphocytidyl-2-C-methyl-D-erythritol kinase (CMK), which belongs to the IspE superfamily, was responsible for the *al4* mutant phenotype (**Figure 2**). *IspE* encodes an intermediate enzyme in the methylerythritol phosphate (MEP) pathway of isoprenoid biosynthesis.

Isoprenoids are the largest group of natural products and are derived from the basic building block isopentenyl diphosphate (IPP) and its allylic isomer dimethylallyl diphosphate (DMAPP) (Hoshino and Gaucher, 2018). IPP and DMAPP are mainly synthesized *via* two pathways: the mevalonate (MVA) pathway and the non-mevalonate pathway, which is also known as the 2-C-methyl-D-erythritol 4-phosphate (MEP) pathway (Rohmer, 1999). The MEP-pathway comprises seven nuclear-encoded plastid-localized enzymes (Zeng and Dehesh, 2021). Previous studies have shown that defects in MEP pathway genes may result in leaf color change phenotype in different plant species.

1-Deoxy-D-xylulose-5-phosphate synthase (DXS) catalyzes the first committed step in the MEP pathway, *Arabidopsis DXS1* knock-out *cla1-1* mutant displayed albino phenotype and can be rescued by the addition of 1-Deoxy-D-Xylulose (DX) (Mandel et al., 1996; Estévez et al., 2000). In tomato, the *DXS1* T-DNA mutant *wls-2297* has a severe deficiency in chlorophylls and carotenoids, leading to an albino phenotype (García-Alcázar et al., 2017). The second step is catalyzed by 1-deoxy-d-xylulose-5-phosphate reductoisomerase (DXR), which transforms 1-deoxy-D-xylulose 5-phosphate (DOXP) to MEP, notably, disruption of DXR genes also results albino phenotype in plants. For example, the *Arabidopsis* T-DNA insertion mutant *dxr* exhibited an albino phenotype and grew purple cotyledons. Further analysis revealed that the *dxr* mutant only developed proplastids, without normal thylakoids (Xing et al., 2010). Taken together, emerging evidence has demonstrated that the MEP pathway is extensively involved in chloroplast development, leading to changes in leaf color.

IspE catalyzes the ATP-dependent conversion of 4-diphosphocytidyl-2-C-methyl-D-erythritol (CDP-ME) to 4-diphosphocytidyl-2-C-methyl-D-erythritol 2-phosphate (CDP-MEP) in the MEP pathway (Rohdich et al., 2000). A defect in IspE activity might decrease the level of cellular metabolites produced by the MEP pathway, such as chlorophylls and carotenoids (**Figure S10**) (Ahn and Pai, 2008). Virus-induced gene silencing of *IspE* in *Nicotiana benthamiana* results in defective biogenesis of both chloroplasts and mitochondria, leading to severe leaf yellowing phenotypes (Ahn and Pai, 2008). The *Arabidopsis* homozygous T-DNA insertion mutant *ispE-1* is albino lethal, and thylakoid development is completely abolished in the *ispE-1* mutant (Hsieh et al., 2008). The *IspE* gene was further characterized in the monocotyledonous model plant rice, and map-based cloning revealed that a missense mutation in *OsIspE* in *gry340* mutant, resulted in a green-revertible yellow leaf phenotype (Chen et al., 2018). The OsIspE protein is localized in the chloroplast (Chen et al., 2018). Further analysis revealed that *OsIspE* altered the expression of other MEP and MVA pathway genes (Chen et al., 2018).

Notably, in the present study, the *ispE* (*al4*) mutant displayed an albino phenotype, which was not completely consistent with *gry340*. A single nucleotide C to T substitution at position 2,554 of the *IspE* coding region was detected in the *gry340* mutant, whereas deletion of 96 bp and the entire exon2 was absent of *IspE* in the *al4* mutant, suggesting that variable expression might lead to different phenotypic effects in *gry340* and *al4* mutants. Defects in leaf color could be attributable to the suppression of expression of chloroplast biosynthetic genes, thus we analyzed the expression patterns of a series of chloroplast-related genes. Results showed that most of genes were significantly reduced in the *al4* compared to WT, such as photosynthetic associated nuclear genes (PhANGs), suggesting that defective chloroplast development in *al4* mutant might be associated with the repression of chloroplast-associated genes (**Figure 6**). We also investigated the expression pattern of mitochondrial genes between WT and al4 mutant, including *cob*, *atp8*, *cox1*, and *nad2* ect, results showed that few genes, such as *cox1* have different expression pattern between *al4* and *gry340* mutants.

Alternative splicing (AS) is an important posttranscriptional process that produces different mRNA isoforms through the selection and utilization of alternative splice sites in the same pre-mRNA (Simpson et al., 2008; Reddy et al., 2013). Several types of AS were identified and classified, including intron retention (IR), alternative 5’ splice site (A5SS), alternative 3’ splice site (A3SS), mutually exclusive exons (MXE), and exon skipping (ES). The excision of introns from a pre-mRNA is directed by special sequences at the intron-exon junctions (Black, 2003), and splicing of pre-mRNA is controlled by a multi-megadalton ribonucleoprotein (RNP) complex, called the spliceosome, which leads to the removal of introns from pre-mRNA and assembly of a mature transcript (Brown and Simpson, 1998; Will and Lührmann, 2011).

The first step in pre-mRNA splicing is the recognition and selection of specific splicing sites. Previous studies have shown that disruption of splice sites commonly results in missing an exon and both flanking introns during the splicing process. For example, a SNP on the splicing site of *CsSEP2*, resulted in the skipping of exon6 and abolishment of the transcriptional activity in *CsSEP2*, leading to perturbed floral and fruit development in cucumber (Wang et al., 2016). A single mutation(G-to-A) at the splice site of intron 5 in *CRS1*, results in exon skipping and perturbed chloroplast development in maize (Wang et al., 2021). In the present study, mutation at the exon-intron junction site caused the spliceosome complex fail to distinguish of the original splice site, leading to 96 bp deletion and entire exon2 was absent in the *al4* mutant, which displayed an albino phenotype (**Figure 3**), therefore the exon skipping occurred during splicing due to the mutation in the splice site.

In conclusion, the present study showed that *OsIspE* is extensively involved in chloroplast biogenesis and development. Furthermore, mutation at the exon-intron junction site in *IspE* resulted in exon skipping and produced truncated *IspE*, eventually exhibiting an albino phenotype. Moreover, the findings of this study suggest that *OsIspE* regulates chloroplast biogenesis and development through coordinated transcription of chloroplast-associated genes, particularly PhANGs.

## Data availability statement

The raw data supporting the conclusions of this article will be made available by the authors, without undue reservation.

## Author contributions

TX and ZZ conceived and designed the study. TX and JZ conduct the most of experiments and analyzed the data. YL performed the Mutmap+ analysis. QZ, WL, YZ, MW, TC, and DD helped with the data interpretation. WW and ZZ analyzed the data and drafted the manuscript. ZZ supervised the study. All authors contributed to the article and approved the submitted version.

## Funding

This study was supported by the Key-Area Research and Development Program of Guangdong Province (2018B020202012), and the Natural Science Foundation of China (31671645).

## Conflict of interest

The authors declare that the research was conducted in the absence of any commercial or financial relationships that could be construed as a potential conflict of interest.

## Publisher’s note

All claims expressed in this article are solely those of the authors and do not necessarily represent those of their affiliated organizations, or those of the publisher, the editors and the reviewers. Any product that may be evaluated in this article, or claim that may be made by its manufacturer, is not guaranteed or endorsed by the publisher.

## Abbreviations

MEP, Methylerythritol phosphate; MVA, Mevalonic acid; *al4*, *albino leaf 4*; PPR: Pentatricopeptide repeat protein; IPP: Isopentenyl diphosphate; DMAPP, Dimethylallyl diphosphate; CMK, 4-diphosphocytidyl-2-C-methyl-D-erythritol kinase; EMS, Ethyl methyl sulfonate; Chla/Chlb, Chlorophyll a/b; Car, Carotenoid; DAP, day after germination; TEM, Transmission electron microscopy; PhANGs, photosynthetic associated nuclear genes; GFP, Green fluorescent protein.
