# Supplementary material for: Exon skipping in IspE Gene is associated with abnormal chloroplast development in rice albino leaf 4 mutant
Source: Front Plant Sci. 2022 Nov 8;13:986678. doi: 10.3389/fpls.2022.986678 (PMC9678938; doi:10.3389/fpls.2022.986678)
Supplement: Supplementary file 1 [file DataSheet_1.docx]

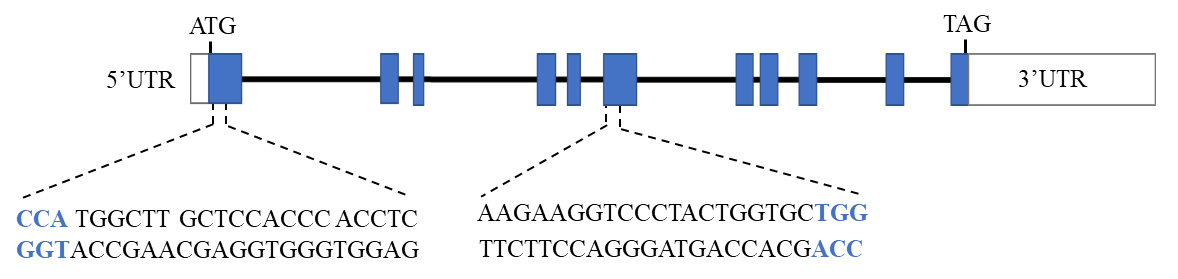


**Figure S1.** A schematic representation of knockout target sites of *OsIspE*


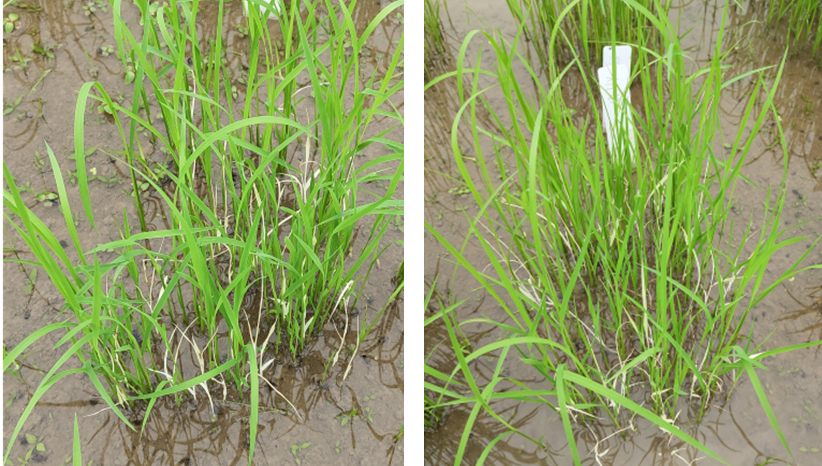


**Figure S2.** M_3_ phenotype of *IspE* heterozygous


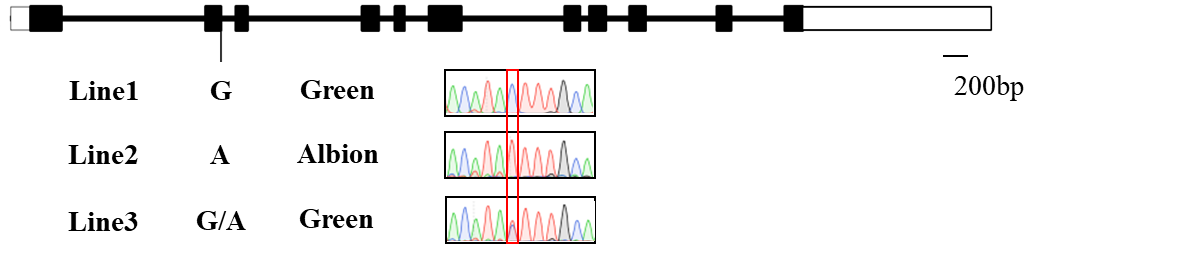


**Figure S3.** Sanger sequence of M_4_ plants

**
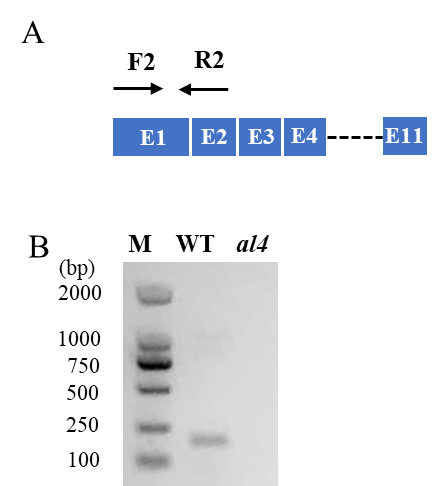
**

**Figure S4.** RT-PCR of *OsIspE* in the wild type and *al4* mutant with the specific primers. **A-B** RT-PCR bands of *OsIspE* in the wild type and *al4* mutant with the specific primers indicated in Fig A.

**Figure S5.** A single nucleotide mutation in *al4* and *gry340* mutants


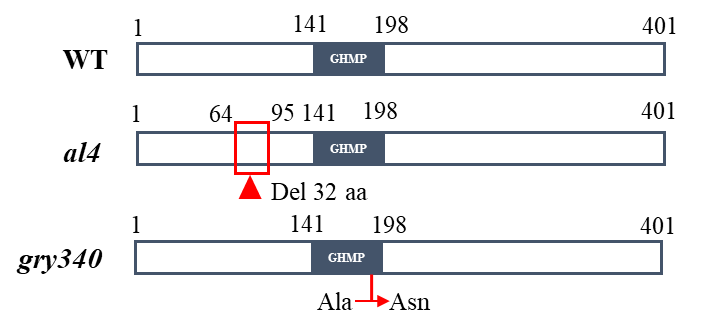


**Figure S6.** Protein comparison between the *al4* and gry340 mutants

**Figure S7.** RT-qPCR analysis of *OsIspE* expression in WT and *al4*

**
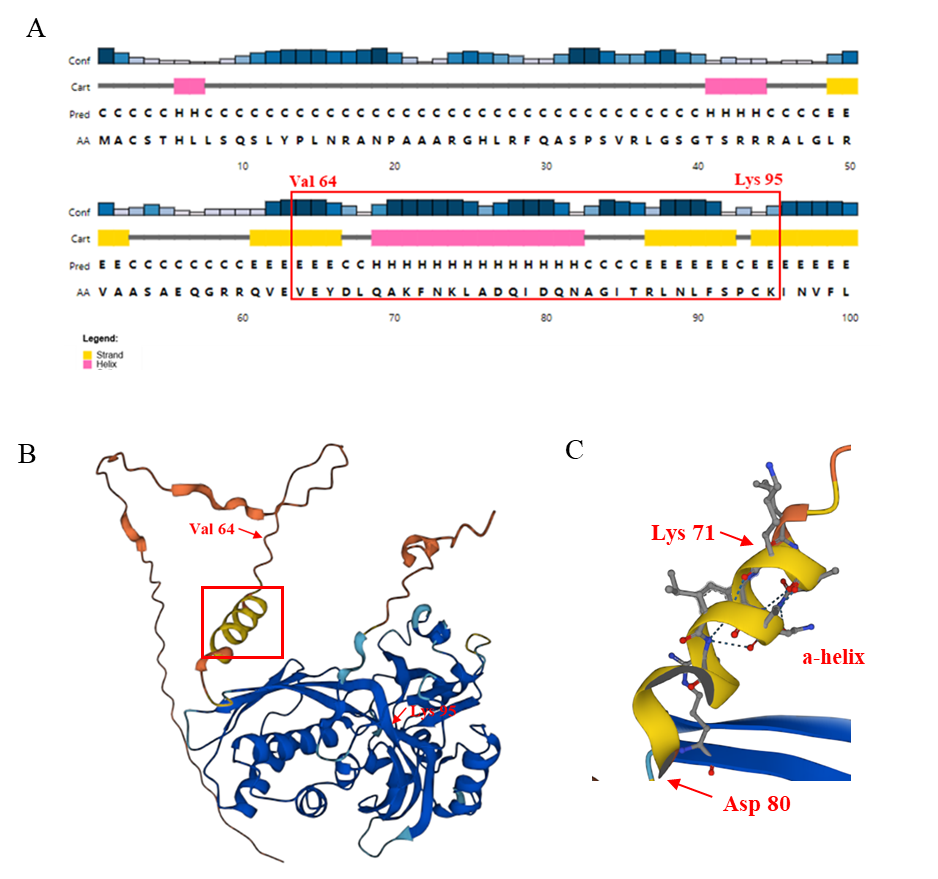
**

**Figure S8.** Protein structure protein of OsIspE. (A) Protein sequence of N terminus of protein; (B) Protein structure protein of OsIspE with SWISS MODEL; (C) OsIspE Exon2 contains α-helix

**Figure S9.** Expressions analysis of mitochondrial genes in the wild type and the *al4* mutant by RT-qPCR

**
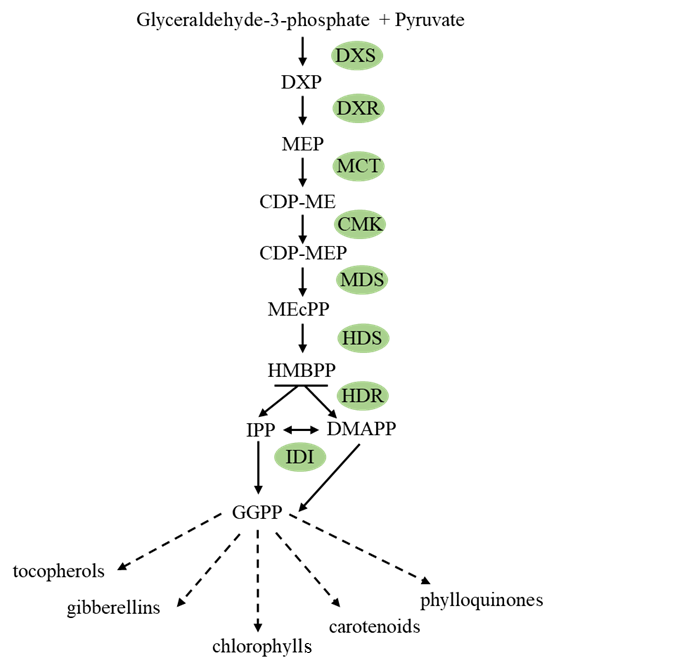
**

**Figure S10.** MEP pathway in plants

**Supplemental Table 1. Primers used for RT-qPCR**

| Primer name | Forward (from 5’ to 3’) | Reverse (from 5’ to 3’) |
| --- | --- | --- |
| OsUBQ | TGGTCAGTAATCAGCCAGTTTGG | GCACCACAAATACTTGACGAACAG |
| IspE-QRT | CGGACCAAATTGACCAGAATGC | ACACCTGCTACATTGGTTGAC |
| IspE-RT-1 | ATGGCTTGCTCCACCCAC | TCAGTCAGAAACTGATGC |
| IspE-RT-2 | TCCAGGCTTCTCCCAGTGTGA | AGGTTCAGCCGTGTAATCCCA |
| HAP3A | TCTGTTAAGGAAGAACCCAC | TAGATTTGTGCCACCTGATA |
| HAP3B | AACTGCAAAGGCTGGTGATGGCTCT | TACATCTGAGAAGCAGCCTTGGCTC |
| Cab1R | AGATGGGTTTAGTGCGACGAG | TTTGGGATCGAGGGAGTATTT |
| Cao1 | CAGAATCCAATGCCCTTAC | AGTATCCTTGAAGCCCAGA |
| HEMA1 | CGCTATTTCTGATGCTATGGGT | TCTTGGGTGATGATTGTTTGG |
| psbO | GCTCTACCGGCTACGACAAC | TGACATCCTTGGGCACCTT |
| psbP | AAGACAGATTCCGAGGGTGG | TGATTCGCTAGGGATTAAAGAG |
| lhcb2 | CCCCATCGAGAACCTCTTC | CGGTGCGTGGCTACTACAA |
| psaE | CCGCCAAGCCGCCTCCCATT | AGCTCGACGACGATCCATCC |
| psaD | CCGCTCCAAGTACAAGATCA | AAGAGCAGCCTGACAGATGA |
| rbcS | TGAGGGCATCAAGAAGTT | CGATGATACGGACAAAGG |
| psaB | GAGCAATATCGGTCAGCCACA | ACCACTCAAGGAGCGGGAAC |
| psaA | TTAGAAATCCGCCAATCCA | TGCTAGGCTCTACAACCATT |
| psbA | ACCCTCATTAGCAGATTCGT | GATTGTATTCCAGGCAGAGC |
| petA | TGCCATTTAGCGAATAAGCC | CCACATTCAACCCTCCCTTT |
| atpA | TATCGGTCAAAGAGCATC | CGTATAAGGAGCGAGGTA |
| rps2 | GAGATGATAGAAGCGGGAGTT | TAACATAATGACAACGAGCC |
| cob | GTTCTTTGCCATAACGCC | CTAAGAGACTGATCCGGT |
| cox1 | GGCTGTTGAACAGAATCC | GATAGCTGGAAGTTCTCC |
| nad2 | TTATGTGATCGCAGCATC | GGAAATGCACCTAAGATC |
| nad4 | TGTTCGGATGGGTGTTCA | TTGCCATGTTGCACTAAG |
| nad5 | TGCAATGTTACTTGGTTC | AGAAAACTGCTCACTAAC |
| atp6 | TTCGCTTGGACTATGCTA | TACACCTAATTCCAGACC |
| atp8 | GAGGAATGGAGAGACAGA | TGTGGAACATGTGTGAGC |

**Supplemental Table 2. Primers used for vector construction and phenotype detection**

| Aplication | Primer name | Primer sequence (from 5’ to 3’) |
| --- | --- | --- |
| *OsIspE*-Cas9 vector | AL4-OsU6a-F | gccgAGGTGGGTGGAGCAAGCCA |
|  | AL4-OsU6a-R | aaacTGGCTTGCTCCACCCACCT |
|  | AL4-OsU3-F | ggcaAGAAGGTCCCTACTGGTGC |
|  | AL4-OsU3-R | aaacGCACCAGTAGGGACCTTCT |
| pRTV-OsIspE-cGFP vector | AL4-cGFP-F | ACGAGCTCATGGCTTGCTCCACCCAC |
|  | AL4-cGFP-R | CCAAGCTTGTCAGAAACTGATGCTACC |
| P1305-OsIspE-cGFP vector | P1305-AL4-GFP-F | CAGCCCAGATCAACTAGTATGGCTTGCTCCACCCAC |
|  | P1305-AL4-GFP-R | CTTGCTCACCATGGATCCGTCAGAAACTGATGCTACC |
| Mutmap+ and SNP detection | IspE-F | TAAATTCCTGATCGCTGCAT |
|  | IspE-R | TCTTAATGATGACACCCCTTG |
| IspE target1 detection | AL4-T1-F | TCCTCTTCGCCTCTGGTAAGC |
|  | AL4-T1-R | TCGCCTCTGAGTTTAGCGTG |
| IspE target2 detection | AL4-T2-F | CGCTTGTCAACCAATGTAGCAG |
|  | AL4-T2-R | ATTCACGGCAATTAGAAGCAG |
|  | Cas9-F | CACCATCTACCACCTGAGAA |
|  | Cas9-F | CGAAGTTGCTCTTGAAGTTG |

**Supplemental Table 3. Segregation ratio of *IspE* heterozygous**

| Lines | M_4_ lines | | | ꭓ^2^ test | p value |
| --- | --- | --- | --- | --- | --- |
|  | Total | Albino | Green | （ꭓ^2^ 0.05=3.84） |  |
| Line 1 | 215 | 55 | 160 |  |  |
|  |  |  |  |  |  |
| Line 2 | 163 | 39 | 124 |  |  |
|  | 378 | 94 | 284 | 0.0035273 | 0.9526 |

**Supplemental Table 4. Information for candidate gene**

| Number | Genotype WT/*al4* | Gene ID | Variation type | Putative function |
| --- | --- | --- | --- | --- |
| SNP1 | G/A | *Os01g0129200* | downstream gene variant | SL1 |
| SNP2 | C/G | *Os01g0676800* | downstream gene variant | Conserved hypothetical protein |
| SNP3 | C/G | *Os01g0676900* | upstream gene variant | OsSPEAR3 |
| SNP4 | A/G | *Os01g0769900* | intron variant | Similar to PTAC12 |
| SNP5 | A/G | *Os01g0770000* | downstream gene variant | wax synthase |
| SNP6 | C/T | *Os01g0778400* | upstream gene variant | OsAGP19 |
| SNP7 | T/C | *Os01g0778700* | intron variant | transmembrane protein 49 |
| SNP8 | C/T | *Os01g0788400* | downstream gene variant | OsPME6 |
| SNP9 | C/T | *Os01g0788451* | upstream gene variant | Hypothetical protein |
| SNP10 | C/T | *Os01g0802100* | intron variant | 4-diphosphocytidyl-2-C-methyl-D-erythritol kinase |
| SNP11 | C/T | *Os02g0100200* | upstream gene variant | Steroid nuclear receptor |
| SNP12 | C/T | *Os02g0100250* | downstream gene variant | Hypothetical protein |
| SNP13 | C/T | *Os02g0580500* | upstream gene variant | mitochondrial prohibitin complex protein 1 |
| SNP14 | C/T | *Os02g0580700* | upstream gene variant | Conserved hypothetical protein |
| SNP15 | C/T | *Os02g0611200* | upstream gene variant | SAMDC2 |
| SNP16 | C/T | *Os02g0611250* | downstream gene variant | Hypothetical gene |
| SNP17 | A/T | *Os02g0655600* | upstream gene variant | serine esterase family protein |
| SNP18 | C/T | *Os03g0149300* | downstream gene variant | Protein of unknown function DUF6 |
| SNP19 | C/T | *Os03g0165600* | 3' UTR variant | Peptidase C19 |
| SNP20 | A/G | *Os03g0242100* | intron variant | cell cycle checkpoint protein RAD17 |
| SNP21 | G/A | *Os03g0304400* | downstream gene variant | Zinc finger |
| SNP22 | C/T | *Os04g0394100* | downstream gene variant | Similar to H0718E12.3 protein |
| SNP23 | C/T | *Os04g0394200* | intron variant | Similar to 2-oxoglutarate dehydrogenase E2 subunit |
| SNP24 | C/T | *Os04g0394300* | downstream gene variant | OsC3H27 |
| SNP25 | G/A | *Os05g0371600* | downstream gene variant | OsRLCK184 |
| SNP26 | G/C | *Os05g0390500* | upstream gene variant | BRCT domain containing protein |
| SNP27 | T/G | *Os06g0182300* | missense variant | GSL5 |
| SNP28 | C/T | *Os07g0617500* | upstream gene variant | Plant disease resistance response protein family protein |
| SNP29 | G/A | *Os08g0441600* | upstream gene variant | chorismate mutase |
| SNP30 | G/A | *Os09g0287300* | upstream gene variant | phosphoglycerate mutase |
| SNP31 | G/A | *Os09g0287500* | upstream gene variant | DUF565 domain containing protein |
| SNP32 | C/T | *Os10g0558125* | upstream gene variant | matrixin family protein |
| SNP33 | C/T | *Os10g0558301* | downstream gene variant | Hypothetical gene |
| SNP34 | A/T | *Os11g0503400* | upstream gene variant | Conserved hypothetical protein |
| SNP35 | C/T | *Os11g0531700* | intron variant | NUDIX hydrolase domain containing protein |
| SNP36 | C/T | *Os11g0532000* | downstream gene variant | Conserved hypothetical protein |
| SNP37 | G/T | *Os12g0267400* | upstream gene variant | Hypothetical protein |
| SNP38 | G/T | *Os12g0267500* | downstream gene variant | Conserved hypothetical protein |
| SNP39 | C/T | *Os12g0448900* | synonymous variant | PIOX |
| SNP40 | A/C | *-* | intergenic variant | - |
| SNP41 | C/T | - | intergenic variant | - |
